# The moderating role of athletic mental energy in the relationship between pessimism and burnout among athletes competing in university sports competitions

**DOI:** 10.3389/fpsyg.2026.1968065

**Published:** 2026-10-09

**Authors:** Ferhat Çifçi, Ersin Ballıkaya, Abdurrahman Demir

**Affiliations:** 1 School of Physical Education and Sports, Dicle University, Diyarbakır, Türkiye; 2 Hüseyin Güvercin Secondary School, Mersin, Türkiye; 3 School of Physical Education and Sports, Osmaniye Korkut Ata University, Osmaniye, Türkiye

**Keywords:** athlete burnout, athletic mental energy, athletics, pessimism, university athletes

## Abstract

**Purpose:**

This study aimed to examine the moderating role of athletic mental energy in the relationship between pessimism and athlete burnout among athletes participating in the Turkish University Athletics Championships.

**Methods:**

A correlational research design was employed in this study. The sample consisted of 235 track and field athletes, including 85 female and 150 male athletes. Data were collected using the Athletic Mental Energy Scale, the Sport Pessimism Scale, the Athlete Burnout Scale, and a Personal Information Form. Pearson’s correlation analysis and moderation analysis using Hayes’ PROCESS macro were conducted to analyze the data.

**Results:**

The research findings revealed a significant positive association between pessimism and athlete burnout (*r* = 0.334, *p* < 0.01). Athlete burnout was also significantly and negatively associated with the vigor (*r* = −0.171, *p* < 0.01) and motivation (*r* = −0.159, *p* < 0.05) dimensions of athletic mental energy. Furthermore, athletic mental energy (*β* = −0.940, Δ*R*^2^ = 0.018, *p* < 0.05) exerted a significant negative moderating effect on the relationship between pessimism and athlete burnout. Among its subdimensions, confidence (*β* = −1.002, Δ*R*^2^ = 0.029, *p* < 0.05), tirelessness (*β* = −0.914, Δ*R*^2^ = 0.031, *p* < 0.01), and concentration (*β* = −1.096, Δ*R*^2^ = 0.039, *p* < 0.01) also demonstrated significant negative moderating effects.

**Conclusion:**

The findings indicate that the effect of pessimism on athlete burnout varies according to the levels of athletic mental energy, self-confidence, tirelessness, and concentration. Specifically, as levels of athletic mental energy, self-confidence, tirelessness, and concentration increased, the effect of pessimism on athlete burnout progressively weakened. Future research should investigate the long-term effects of athletic mental energy on pessimism and athlete burnout using longitudinal study designs.

## 1 Introduction

Athletes are exposed to prolonged and intensive training loads in pursuit of high performance and competitive success, reflecting the inherently demanding nature of the sporting environment (Rice et al., 2016). Beyond physical training, psychological factors play a crucial role in athletes’ ability to perform at a high level (Singh et al., 2024). Researchers in the fields of sport and psychology have reported that psychological factors contribute positively to athletes’ pursuit of their goals and to their experiences in both sport and everyday life (Weinberg and Gould, 2019). However, demanding training and competitive environments, along with high performance expectations, can trigger or exacerbate a range of psychological difficulties among athletes. Fu et al. (2026) reveal that athletes can experience physical and psychological exhaustion due to overtraining and inadequate recovery, and that this may be associated with depressive symptoms. Similarly, Kuzu and Çelik (2024) suggested that setting achievement-oriented goals, coupled with the pressure to win, may lead to heightened anxiety and stress among athletes, which may, in the long term, contribute to burnout and negatively affect their psychological wellbeing.

In the sporting context, intensive training loads, competitive environments, and high performance expectations can increase athletes’ stress levels. When elevated stress persists over an extended period and becomes chronic, it may contribute to the development of burnout, which can have serious adverse consequences (Li et al., 2025). Burnout, which is commonly observed among athletes (Gustafsson et al., 2018), may arise when athletes are unable to cope effectively with chronic stress (Maslach and Leiter, 2016). Athlete burnout is a multidimensional construct characterized by a reduced sense of accomplishment associated with diminished perceptions of competence in athletic performance and sport-related abilities (Gustafsson et al., 2008), sport devaluation accompanied by decreased motivation and interest in sport participation, and physical and emotional exhaustion (Raedeke and Smith, 2001). Research has demonstrated that burnout can have significant consequences for athletes’ sporting lives. Specifically, existing studies have associated athlete burnout with a range of adverse psychological and behavioral outcomes, including reduced psychological wellbeing (Nicholls et al., 2022), diminished motivation (Martinent et al., 2014), depression (De Francisco et al., 2014), difficulties in interpersonal relationships (Isoard-Gautheur et al., 2016), and an increased risk of withdrawal from or dropout from sport (Gould and Whitley, 2009).

In addition to physical factors such as training load, performance pressure, and demanding competitive seasons, psychological factors are also associated with athlete burnout. One such factor is pessimism, which, together with optimism, influences how athletes interpret success, failure, and their own performance. Indeed, despite having considerable performance potential, some athletes may exhibit pessimistic thinking by overlooking their positive attributes and strengths (Tekkurşun Demir, 2022). Such thoughts can shape athletes’ behavior by influencing how they appraise situations in actual competitive settings, regulate their emotions, and form expectations about future outcomes (Kurova et al., 2023). Carver et al. (2010) suggested that pessimism is associated with negative emotions such as hopelessness, a tendency to focus on adverse experiences, and expectations of unfavorable outcomes. Literature has shown that optimism and pessimism are interconnected concepts occurring at opposite extremes (TenHouten, 2023), that optimism can function as a protective factor against athlete burnout, and that pessimism contributes to higher levels of emotional exhaustion in athletes (Malán-Ernst et al., 2025). Gaudreau and Blondin (2004) emphasized that optimistic and pessimistic tendencies in sport are associated with athletes’ goal attainment, coping strategies, and emotional states in competitive contexts. In particular, they reported that pessimism is associated with greater use of disengagement-oriented coping strategies, which involve avoiding rather than dealing directly with stressful situations, as well as with greater anger and depression following competition. Similarly, Horozoğlu and Korkmaz (2025) stated that pessimism increases anxiety levels in professional athletes. Yücekaya et al. (2025) also noted that pessimism can be associated with decreased self-confidence, loss of motivation, and concentration problems in athletes, as well as causing them to focus on potential threats and expect negative outcomes, which can generally be related to low performance. This tendency to expect negative outcomes may reduce athletes’ potential athletic performance by preventing them from recognizing opportunities for success (Scheier et al., 1994). Norem and Chang (2002) further argued that pessimism may operate as a self-fulfilling prophecy by increasing stress and impairing performance. In this context, pessimism, conceptualized together with optimism as part of a bipolar structure, may be an important variable associated with athlete burnout. Although the literature indicates that identifying factors of burnout and implementing appropriate planning and training programs to optimize athletic performance may contribute to sustained participation in sport, it also highlights the need to identify moderating variables that may contribute to the prevention and management of burnout and to develop models incorporating these variables (Carlin and de los Fayos Ruiz, 2010; García-Parra et al., 2016; Angosto et al., 2021).

Athletic mental energy is another important variable with the potential to contribute to the regulation and management of athlete burnout. Indeed, athletic performance is influenced not only by physical factors, such as physical fitness, but also by psychological and emotional characteristics (Olmedilla et al., 2019). Lu et al. (2018) reported that athletic mental energy enables athletes to sustain their physical and mental effort during intensive training and competition. They defined athletic mental energy as an athlete’s perceived current state of energy, characterized by the intensity of motivation, confidence, concentration, and mood. Athletic mental energy, which involves the ability to think about a task for extended periods, sustain attention, and resist distractions in order to accomplish a given task (Lykken, 2005), has been found to be negatively associated with athlete burnout and life stress and positively associated with positive mood (Lu et al., 2018). In this context, the multidimensional structure of athletic mental energy, consisting of vigor, confidence, motivation, tirelessness, concentration, and calmness, suggests that different psychological resources may play a role in athletes’ coping with negative thoughts and intense performance demands. For example, confidence may reduce the effects of pessimistic thoughts by supporting athletes’ positive evaluations of their abilities, whereas endurance may support the maintenance of effort in the face of intense performance demands, and concentration may support focusing on the task by controlling distracting factors. Therefore, the different dimensions of athletic mental energy may be considered psychological resources that can change the strength of the relationship between pessimism and burnout. Indeed, research suggests that athletic mental energy is an important psychological resource associated with competitive performance. For example, Shieh et al. (2023) found that mental energy was associated with athletes’ competitive performance and, in particular, predicted the performance of volleyball setters. These findings suggest that athletic mental energy may represent an important positive psychological resource that supports athletic performance. Research suggests that these components are associated with the optimal level of arousal required for high athletic performance among athletes with high levels of athletic mental energy (Pratama et al., 2025; Durand-Bush et al., 2023; Lochbaum et al., 2021; Chuang et al., 2022). The literature further indicates that an important characteristic of successful athletes is their ability to maintain their focus on performance without losing attention (Yarayan et al., 2025), and that possessing sufficient mental energy may be particularly important for performance in sporting activities that require sustained concentration (Lykken, 2005). When considered together, these studies indicate that optimal arousal, attention and mental energy are important psychological characteristics associated with sporting performance. In this regard, the findings suggest that athletic mental energy can be regarded as a psychological resource that supports athletes’ performance. Chiou et al. (2020) reported that higher levels of mental energy may reduce the adverse effects of stress and burnout among athletes. Smith et al. (2016) reported that mental fatigue, which is conceptually opposite to the components of athletic mental energy, particularly vigor, motivation, and concentration, may negatively affect athletes’ technical skills, such as passing and shooting, as well as their reaction time and decision-making abilities. This suggests that mental resources supporting athletes’ cognitive functioning and sporting performance are important and provides a theoretical basis for examining whether athletic mental energy may serve as a potential moderator in the relationship between adverse psychological processes and athlete burnout. Singh et al. (2024) found that athletic mental energy mediated the relationship between competitive performance and psychological wellbeing among athletes participating in different sports, including football, cricket, basketball, and archery. Alongside these studies focusing on athletic performance, examining the relationship between athletic mental energy and athlete burnout is important for understanding the various aspects of the role that mental energy plays in athletes. Indeed, while athletic mental energy is regarded as one of the positive psychological resources that support athletic performance, it may also play a significant role in reducing burnout.

In this context, athletic mental energy, which encompasses positive psychological states such as vigor, confidence, motivation, tirelessness, concentration, and calmness, is associated with high athletic performance. Athletic mental energy encompasses positive psychological resources that may help athletes maintain their wellbeing and has the potential to be a potentially protective resource in managing and regulating adverse psychological outcomes, such as pessimism and burnout, that may arise in response to poor performance. Indeed, recent research suggests that athletic mental energy may help maintain athletes’ wellbeing. Although previous research has linked pessimism to stress, maladaptive coping, negative emotions, and burnout, studies have mainly examined its direct associations with psychological outcomes. Similarly, research on athletic mental energy has primarily focused on its direct relationships with burnout, stress, performance, and psychological wellbeing. However, in the existing literature, there is limited research into whether the relationship between pessimism and athlete burnout varies according to personal psychological resources such as athletic mental energy. In particular, no research has been found on whether athletic mental energy and its components influence the strength of the relationship between pessimism and athlete burnout. Consequently, the existing research gap is not limited merely to examining the relationship between pessimism and athlete burnout; it also extends to clarifying whether this relationship differs depending on the levels of athletic mental energy and its components. From this perspective, while pessimism is treated as a cognitive risk factor and athletic mental energy and its components are evaluated as psychological resources, there is a notable lack of research addressing the relationship of these two constructs with athlete burnout within the same theoretical framework. Thus, treating athletic mental energy as a moderator offers a more comprehensive approach to understanding not only whether a relationship between pessimism and burnout exists, but also whether this relationship varies depending on the level of athletic mental energy. From a theoretical perspective, athletic mental energy may function as a psychological resource that may be associated with athletes’ responses to the negative cognitive and emotional reactions to pessimism. According to the Conservation of Resources Theory (Hobfoll, 1989), the psychological resources individuals possess play an important role in coping with stress, and the depletion of these resources may be associated with negative outcomes such as burnout. In this context, psychological resources such as motivation, confidence, concentration, and attention may support athletes in coping with stress and maintaining their existing resources when faced with negative expectations and stressful experiences. Therefore, athletes with higher levels of athletic mental energy may be better able to utilize such psychological resources, which may contribute to a weaker association between pessimism and burnout. In other words, the association between pessimistic thoughts and burnout may be weaker among athletes with higher levels of athletic mental energy because higher athletic mental energy may support the maintenance of motivation, confidence, and concentration in the face of negative cognitive and emotional processes. Within this framework, the contribution of the present study is to consider athletic mental energy in the sports context not merely as a psychological characteristic associated with burnout, but rather as a psychological resource that may help explain how the relationship between an adverse cognitive tendency, such as pessimism, and burnout may vary in strength. Thus, the present study extends the findings of research suggesting that pessimism is associated with burnout, while providing a framework for assessing the strength of this relationship in conjunction with the psychological resources possessed by athletes. This approach broadens burnout beyond simply considering it as a consequence of negative cognitive processes, to also take into account the psychological conditions underlying these processes. In particular, given that athletic mental energy encompasses attributes such as maintaining self-confidence, concentration, and motivation, it may be linked to athletes’ ability to focus on their sporting tasks and utilize their existing psychological resources when faced with negative expectations and appraisals of failure associated with pessimism. Therefore, it is reasonable to expect that variations in levels of athletic mental energy will influence the strength of the relationship between pessimistic tendencies and burnout. In this regard, the study contributes to broadening current approaches to explaining athlete burnout by adopting a more holistic perspective that considers psychological resources—such as athletic mental energy—alongside cognitive risk factors like pessimism. Furthermore, by focusing on university athletes, this study enables an examination of this relationship within the context of competitive university sports. In this respect, the study goes beyond examining the direct relationship between athletic mental energy and a specific outcome variable, contributing to a better understanding of the potential moderating role of this construct as a psychological resource that can differentiate the strength of the relationship between negative cognitive tendencies like pessimism and burnout. Accordingly, the proposed model assumes that higher levels of pessimism are associated with higher levels of athlete burnout and that athletic mental energy may alter the strength of this association. Within this framework, the present study aimed to examine the moderating role of athletic mental energy in the relationship between pessimism and athlete burnout among athletes participating in the Turkish University Athletics Championships. Based on this aim, we hypothesized that athletic mental energy will negatively moderate the relationship between pessimism and athlete burnout, such that higher levels of athletic mental energy would be associated with a weaker positive relationship between pessimism and athlete burnout.

## 2 Methods

### 2.1 Research design

This study employed a quantitative correlational research design to examine the moderating effect of athletic mental energy on the relationship between pessimism and athlete burnout among athletes participating in university athletics competitions. A correlational research design is a quantitative research approach that focuses on identifying and describing relationships among variables as they naturally occur, without manipulating or intervening in the conditions under investigation (Kaya et al., 2012).

### 2.2 Participants

The study included 235 out of 311 student athletes from 38 universities who participated in the Turkish University Athletics Championship, organized by the Turkish University Sports Federation (TUSF) between May 16 and 18, 2025. Of these participants, 85 were female and 150 were male. Participants were included in the study using a convenience sampling method. This sampling method is a type of sampling where participants are included based on criteria such as ease of data collection, willingness to participate due to low cost, easy accessibility, and availability at a specific time (Karasar, 2015). In this context, the study included athletes who had reached the age of 18 and who participated on a voluntary basis. The data were collected face-to-face in the stands of the athletics arena while the competitions were in progress. After providing the necessary explanations to the athletes, the data collection tools were administered individually to the participants, and they were asked to complete the scales independently. Completing the scales took approximately 10–15 min for the athletes. Their ages ranged from 18 to 28 years, with a mean age of 20.96 years (SD = 2.11). Regarding athletics disciplines, 153 participants (65.1%) competed in running events, 45 (19.1%) in jumping events, and 37 (15.7%) in throwing events. Participants had between 1 and 21 years of sport participation experience, with a mean of 8.20 years (SD = 4.21). In addition, 73 participants (31.1%) had represented the national team on at least one occasion, 103 (43.8%) had achieved competitive success at the national level, and 57 (24.3%) had achieved competitive success at the international level.

### 2.3 Data collection instruments

The following instruments were used to collect data for the study: a Personal Information Form, the Athletic Mental Energy Scale (AMES), the Athlete Burnout Questionnaire (ABQ), and the Athlete Pessimism Scale (APS).

#### 2.3.1 Personal information form

A researcher-developed personal information form was used to collect information on the participants’ demographic and sport-related characteristics. The form consisted of seven items assessing the student-athletes’ gender, age, age at the onset of sport participation, discipline/event (e.g., running events/100 m, jumping events/long jump, throwing events/shot put), achievement at the national level, achievement at the international level, and national team representation.

#### 2.3.2 Athletic mental energy scale

The Athletic Mental Energy Scale (AMES), developed by Lu et al. (2018) and validated for use in a Turkish sample by Yıldız et al. (2020), was used to assess participants’ perceptions of their athletic mental energy. The scale consists of 18 items rated on a 6-point Likert scale and comprises six subscales: Vigor, confidence, motivation, tirelessness, concentration, and calmness. Each of the six sub-dimensions on the scale consists of three items. Higher scores indicate higher levels of athletic mental energy. The construct validity of the Turkish version was examined using first- and second-order confirmatory factor analyses, and acceptable fit indices were obtained for both models (first-order CFA: χ^2^/df = 2.09, RMSEA = 0.062, SRMR = 0.053, GFI = 0.91, CFI = 0.95; second-order CFA: χ^2^/df = 2.60, RMSEA = 0.075, SRMR = 0.064, GFI = 0.90, CFI = 0.92). For the 18-item Turkish version of the scale, Cronbach’s alpha was reported as 0.91 for the total scale and as 0.79, 0.78, 0.79, 0.83, 0.78, and 0.80 for the six subscales, respectively. In the present study, Cronbach’s alpha was 0.93 for the total scale and 0.71, 0.80, 0.82, 0.83, 0.74, and 0.75 for the six subscales, respectively. In this study, the Cronbach’s alpha coefficient was used to evaluate reliability. Cronbach’s alpha was chosen because it is a commonly used coefficient for evaluating internal consistency between items and reporting the reliability of scales.

#### 2.3.3 Athlete Pessımısm Scale

The Athlete Pessimism Subscale of the Athlete Optimism and Pessimism Scale, developed by Tekkurşun Demir (2022), was used to assess participants’ levels of pessimism in the sport context. The optimism and pessimism subscales can be administered independently. The pessimism subscale consists of nine items rated on a 5-point Likert scale. Higher scores indicate higher levels of pessimism. Specifically, higher scores reflect a stronger belief that past negative sport experiences will recur and that repeated poor performance will ultimately result in failure in one’s sport. The construct validity of the Turkish version of the scale was examined using exploratory and confirmatory factor analyses. The results of the analyses confirmed the scale’s construct validity and showed that the model exhibited acceptable fit indices following confirmatory factor analysis (χ^2^/df = 3.49; RMSEA = 0.09; CFI = 0.98). Tekkurşun Demir (2022) reported a Cronbach’s alpha of 0.90 for the pessimism subscale. In the present study, Cronbach’s alpha was 0.94.

#### 2.3.4 Athlete burnout questionnaire

The Athlete Burnout Questionnaire (ABQ), developed by Raedeke and Smith (2001) and validated for use in a Turkish sample by Kelecek et al. (2016), was used to assess participants’ levels of burnout in the sport context. The ABQ consists of 13 items rated on a 5-point Likert scale and comprises three subscales: Emotional/Physical Exhaustion, Reduced Sense of Accomplishment, and Sport Devaluation. Higher mean scores indicate higher levels of athlete burnout. In the Turkish adaptation of the scale, construct validity was examined using confirmatory factor analysis, and the obtained fit indices were determined to be at an acceptable level (χ^2^/sd = 2.81, RMSEA = 0.06, SRMR = 0.05, CFI = 0.96, GFI = 0.92). Furthermore, concurrent and convergent validity analyses revealed that the scale is a valid measurement tool. In this study, the internal consistency of the scale sub-dimensions was evaluated, and the Cronbach’s alpha internal consistency coefficient for the Reduced Sense of Accomplishment subscale was found to be 0.57. Considering that the low internal consistency in this sub-dimension may increase the measurement error, instead of interpreting the sub-dimensions separately, the total scale score was used to evaluate athlete burnout as a general construct. Cronbach’s alpha for the total scale in the present study was 0.91.

### 2.4 Data analysis

Prior to conducting the basic statistical analyses, the data set was examined in terms of accuracy, missing data and inclusion criteria. In this context, a total of five data points were excluded from the analysis as they did not meet the inclusion criteria (e.g., being under 18 years of age; *n* = 3), were incompletely filled in or had been answered carelessly. Descriptive statistics were first examined to summarize the study data. Before the main analyses, the assumptions of normality and multicollinearity were assessed. The skewness and kurtosis values were used to assess whether the data were normally distributed. The skewness and kurtosis values for the total score of the athletic mental energy scale were determined as −0.203 and −0.582, respectively; for the athlete burnout scale score as 0.830 and 0.145; for the pessimism scale score as 0.523 and −0.903. The skewness values for the subdimensions of athletic mental energy—vigor, confidence, motivation, tireless, concentration, and calm—were found to be −0.681, −0.296, −0.976, −0.020, 0.031, and −0.484, respectively; and the kurtosis values were found to be −0.265, −0.703, 0.288, −0.883, −0.802, and −0.233, respectively. It was determined that the skewness and kurtosis values of the data fell within the acceptable range of −1 to +1. Again, to evaluate the multicollinearity problem, VIF and tolerance values were examined; it was determined that VIF values ranged from 1.31–4.01, and tolerance values ranged from 0.249–0.761. The fact that the VIF values are below 5 and the tolerance values are above 0.20 indicates that there is no issue of multicollinearity amongst the variables. Furthermore, outliers in the dataset were assessed using Mahalanobis distance (χ^2^/df = 14.067, *p* = 0.05), and 18 data points identified as outliers were excluded from the analysis. Following data manipulation and assumption checks, data from 23 out of 258 participants were excluded from the analysis, and the final analyses were performed using data from 235 participants. The relationships among the study variables were examined using Pearson correlation analysis. To examine the moderating effect of athletic mental energy on the relationship between pessimism and athlete burnout, the PROCESS Macro Model 1 for SPSS, developed by Hayes (2017), was used. Moderation analyses were conducted using multiple linear regression models that included the interaction term between the predictor and moderator variables. To prevent the risk of multicollinearity and to ensure the interpretability of regression coefficients during the analysis process, continuous variables were subjected to mean centering before the analysis. In this study, a percentage bootstrap method based on a 95% confidence interval and 5,000 resamples was used to evaluate the reliability of moderation effects. When a significant interaction effect was identified, simple slope analysis was performed to facilitate interpretation of the moderating effect. In simple slope analysis, the relationships between the independent and dependent variables were examined at the mean, one standard deviation below the mean (−1 SD), and one standard deviation above the mean (+1 SD) levels for each moderating variable. The level of statistical significance was set at *p* < 0.05 for all analyses.

## 3 Results

As shown in Table 1, the correlation analysis indicated that pessimism in the sport context was positively associated with athlete burnout (*r* = 0.334, *p* < 0.01). Athlete burnout was significantly and negatively associated with the vigor (*r* = −0.171, *p* < 0.01) and motivation (*r* = −0.159, *p* < 0.05) dimensions of athletic mental energy. However, no statistically significant associations were found between athlete burnout and confidence, tirelessness, concentration, calmness, or total athletic mental energy.

**Table 1:** Correlation analysis results between all study variables.

Variables	1	2	3	4	5	6	7	8	9
1. APS	–								
2. Vigor	0.045	–							
3. Confidence	0.240**	0.705**	–						
4. Motivation	−0.016	0.815**	0.631**	–					
5. Tireless	0.359**	0.628**	0.710**	0.516**	–				
6. Concentration	0.371**	0.523**	0.697**	0.452**	0.728**	–			
7. Calm	0.175**	0.619**	0.601**	0.580**	0.629**	0.613**	–		
8. AME	0.245**	0.860**	0.872**	0.787**	0.853**	0.821**	0.804**	–	
9. Burnout	0.334**	−0.171**	−0.031	−0.159*	−0.048	0.085	0.026	−0.056	–
Mean	22.69	13.63	12.52	14.25	11.30	11.38	12.86	75.94	2.60
SD	10.81	3.26	3.58	3.20	3.87	3.61	3.26	17.33	0.93

APS, Athlete Pessimism Scale; AME, Athletic Mental Energy; *p < 0.05; **p < 0.01.

Table 2 presents the moderating effects of athletic mental energy on the relationship between pessimism and athlete burnout. The results indicated that pessimism was positively associated with athlete burnout [*β* = 0.370, SE = 0.005, *t* = 5.869, *p* < 0.01, 95% CI (0.021, 0.042)], whereas athletic mental energy was negatively associated with athlete burnout [*β* = −0.147, SE = 0.003, *t* = −2.330, *p* < 0.01, 95% CI (−0.015, −0.001)]. Furthermore, total athletic mental energy [*β* = −0.940, SE = −0.000, *t* = −2.217, Δ*R*^2^ = 0.018, *p* < 0.05, 95% CI (0.001, 0.000)] and the confidence [*β* = −1.002, SE = 0.002, *t* = −2.804, Δ*R*^2^ = 0.029, *p* < 0.05, 95% CI (−0.008, −0.001)], tirelessness [*β* = −0.914, SE = 0.001, *t* = −2.948, Δ*R*^2^ = 0.031, *p* < 0.01, 95% CI (−0.007, −0.001)], and concentration [*β* = −1.096, SE = 0.002, *t* = −3.269, Δ*R*^2^ = 0.039, *p* < 0.01, 95% CI (−0.008, −0.002)] subscales had significant negative moderating effects on the relationship between pessimism and athlete burnout. In contrast, the vigor [*β* = −0.699, SE = 0.002, *t* = −1.707, Δ*R*^2^ = 0.011, *p* > 0.05, 95% CI (−0.007, 0.001)], motivation [*β* = −0.239, SE = 0.002, *t* = −0.561, Δ*R*^2^ = 0.001, *p* > 0.05, 95% CI (−0.005, 0.003)], and calmness [*β* = −0.050, SE = 0.002, *t* = −0.135, Δ*R*^2^ = 0.000, *p* > 0.05, 95% CI (−0.004, 0.003)] subscales did not significantly moderate this relationship. Although the study’s findings revealed no significant correlation between total athletic mental energy and athlete burnout (*r* = −0.056, *p* > 0.05), it was determined that athletic mental energy exerted a significant moderating effect on the relationship between pessimism and athlete burnout. This finding suggests that, independently of any direct relationship between athletic mental energy and burnout, the strength of the relationship between pessimism and burnout may vary depending on the level of athletic mental energy.

**Table 2:** Summary results of the moderating effects.

Variables	Step 1: direct effect			Step 2: indirect effect			
	β	*t*-value	*R* ^2^	β	*t*-value	*R* ^2^	∆ *R*^2^
APS	0.005	5.641**	0.146**	0.956	6.643**	0.157**	0.011
Vigor	−0.186	−3.065**		0.107	0.587		
APS × vigor				−0.699	−1.707		
APS	0.363	5.731**	0.125**	1.174	3.967**	0.154**	0.029**
Confidence	−0.118	−1.868		0.282	1.812		
APS × confidence				−1.002	−2.804**		
APS	0.332	5.435	0.135**	0.549	1.402	0.137**	0.001
Motivation	−0.154	−2.526		−0.057	−0.308		
APS × motivation				−0.239	−0.561		
APS	0.403	6.195	0.144**	1.063	4.567	0.175**	0.031**
Tireless	−0.192	−2.954		0.233	1.441		
APS × Tireless				−0.914	−2.948		
APS	0.351	5.276	0.114**	1.169	4.523	0.153**	0.039**
CONCENTRATION	−0.046	−0.687		0.417	2.676		
APS × concentration				−1.096	−3.269		
APS	0.340	5.416	0.113**	0.382	1.224	0.113**	0.000
Calm	−0.034	−0.539		−0.014	−0.087		
APS × calm				−0.050	−0.135		
APS	0.370	5.869	0.132**	1.150	3.220	0.150**	0.018*
AME	−0.147	−2.330		0.204	1.198		
APS × AME				−0.940	−2.217		

APS, Athlete Pessimism Scale; AME, Athletic Mental Energy; *p < 0.05; **p < 0.01.

In the present study, to understand how the moderating effect changes across different conditions, it is necessary to examine the results of the slope analysis showing low, moderate, and high levels of the moderating variable. Figure 1 shows the effects on the relationship between pessimism and athlete burnout when the moderating variable, athletic mental energy, is at low (*β* = 0.054, *p* < 0.01), moderate (*β* = 0.038, *p* < 0.01), and high levels (*β* = 0.025, *p* < 0.01). The findings indicated that the moderating effect was significant at all three levels of athletic mental energy: low, moderate, and high. The steeper slope observed when the moderating variable was low indicates that the positive relationship between pessimism and athlete burnout became stronger. A closer examination of the slopes shows that as athletic mental energy scores increased, its moderating effect on the relationship between pessimism and athlete burnout also increased. In other words, as the level of athletic mental energy increased, the effect of pessimism on athlete burnout gradually weakened.

**Figure 1:**
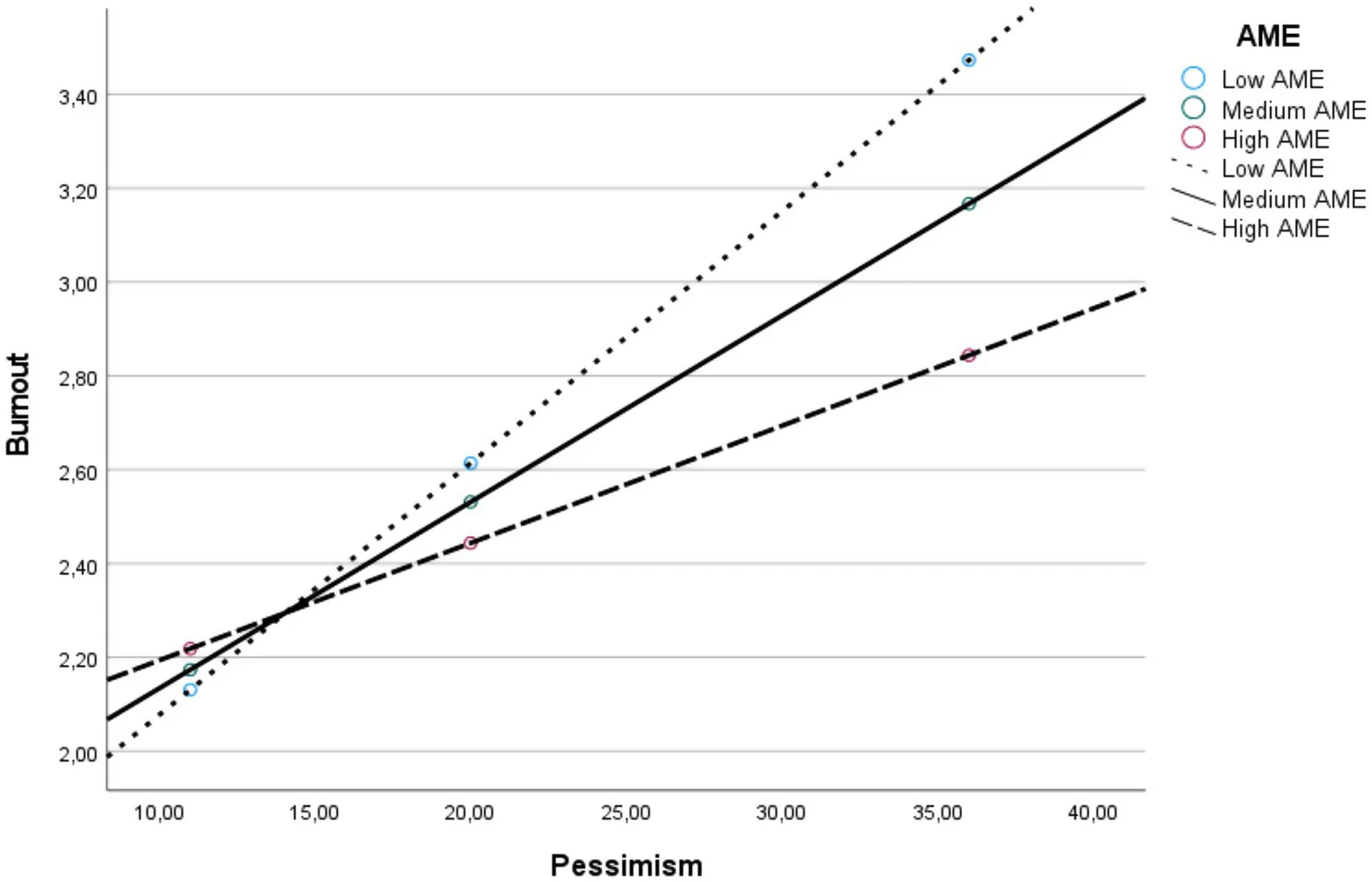
The moderating effects of total athletic mental energy on the relationship between pessimism and burnout.

The simple slope graphs for the confidence, tirelessness, and concentration subscales of athletic mental energy were found to be similar. The moderating effect on the relationship between pessimism and athlete burnout was found to be significant when confidence was low (*β* = 0.063, *p* < 0.01), moderate (*β* = 0.039, *p* < 0.01), and high (*β* = 0.020, *p* < 0.01). Similarly, the moderating effect was statistically significant when tirelessness was low (*β* = 0.062, *p* < 0.01), moderate (*β* = 0.041, *p* < 0.01), and high (*β* = 0.024, *p* < 0.01), as well as when concentration was low (*β* = 0.063, *p* < 0.01), moderate (*β* = 0.042, *p* < 0.01), and high (*β* = 0.021, *p* < 0.01).

## 4 Discussion and conclusion

The present study examined whether athletic mental energy plays a moderating role in the prediction of athlete burnout by pessimism among athletes participating in university sports competitions. The findings revealed that pessimism was positively associated with athlete burnout, whereas athletic mental energy was negatively associated with athlete burnout, and that both variables were significant predictors of athlete burnout. The results further showed that athletic mental energy and its confidence, tirelessness, and concentration components played a moderating role in this relationship. As athletes’ levels of pessimism increased, their levels of burnout also increased; however, as levels of athletic mental energy, confidence, tirelessness, and concentration increased, the adverse effect of pessimism on athlete burnout weakened. However, it was determined that the vigor, motivation, and calmness components of athletic mental energy did not exert a moderating effect on the model. These findings suggest that not all components of athletic mental energy may demonstrate a moderating function to the same extent in the relationship between pessimism and athlete burnout. In the present study, the fact that the components of confidence, tirelessness and concentration in particular moderate the relationship between pessimism and burnout suggests that these components of athletic mental energy may play a more significant role in understanding the strength of this relationship.

The findings of the present study indicate a positive association between pessimism and athlete burnout in the sport context, suggesting that as athletes’ levels of pessimism increase, their levels of athlete burnout also increase. This finding is consistent with previous studies reporting the adverse psychological effects of pessimism on athletes. For example, Astuti et al. (2024), in their study of athletes from different sports disciplines, reported that optimism plays a positive role in psychological functioning, whereas pessimism, a psychological tendency related to but opposite to optimism, is associated with the use of less effective strategies for coping with stress and higher levels of adverse psychological outcomes. Tutte-Vallarino et al. (2022) reported that pessimism was positively associated with the likelihood of experiencing burnout among athletes and that high levels of pessimism increased the likelihood of developing burnout syndrome. Angosto et al. (2021) reported that optimism was negatively associated with both stress and burnout. Chen et al. (2008) similarly reported that optimism was negatively associated with burnout and sport devaluation. Likewise, Gustafsson and Skoog (2012) found that optimistic athletes experienced lower levels of emotional/physical exhaustion and sport devaluation. Although this study did not directly examine pessimism, given that optimism and pessimism are related but opposing psychological orientations, this finding may be considered consistent with the positive association between pessimism and athlete burnout identified in the present study. Taken together, these studies suggest that psychological orientations such as optimism and pessimism may be associated with athlete burnout. However, Rentz (2011), in a study of young soccer players, reported that although optimistic athletes experienced lower levels of burnout, no significant relationship was found between pessimism and burnout. The findings of the present study differ from those reported by Rentz (2011). This difference may be attributable to the characteristics of the sample, sport discipline, and age. Overall, it may be suggested that higher levels of pessimism in the sports environment are associated with higher levels of athlete burnout.

The present study found that athlete burnout was negatively associated with the vigor and motivation dimensions of athletic mental energy. Accordingly, higher levels of vigor and motivation were associated with lower levels of athlete burnout. Chiou et al. (2020) reported that athletes with higher levels of vigor exerted greater effort to cope with sport-related stressors and consequently experienced lower levels of burnout than their counterparts. Lochbaum et al. (2021) reported that athletes’ performance is associated with their level of vigor and that possessing a high level of vigor is important for competitive performance. Similarly, Chuang et al. (2022) state that athletic performance is directly linked to emotional components of mental energy, such as vigor. Brydon et al. (2009) found that more optimistic individuals were able to maintain higher levels of vigor and cope more effectively with stressful situations. This finding suggests that vigor may play a protective role against burnout by supporting athletes’ capacity to cope with stress. This finding suggests that vigor may be a potentially protective resource against burnout by supporting athletes’ capacity to cope with stress.

The negative association identified between motivation and athlete burnout in the present study highlights the need to further consider the potential role of this component of athletic mental energy in burnout. Lu et al. (2018) reported that athletes with lower levels of athletic mental energy also had lower levels of motivation. Graña et al. (2021) reported that motivation was a significant predictor of burnout. Accordingly, athletes’ levels of motivation may play an important role in athlete burnout. In their review, Li et al. (2013) reported that burnout decreased as motivation increased, whereas amotivation increased burnout. Kelecek et al. (2022) reported a positive association between athlete burnout and amotivation. Chiou et al. (2020), reported that athletes with high levels of mental energy and motivation have a greater capacity to exert effort in coping with physical, psychological and social/environmental demands. These findings support the results of the present study, which indicate that athletes are less likely to experience burnout as their levels of motivation increase. This situation may enable highly motivated athletes to be more resilient to burnout.

The findings of the present study indicated that athlete burnout was not significantly associated with overall athletic mental energy or with its confidence, tirelessness, concentration, and calmness dimensions. These findings differ from those reported in some previous studies. For example, Öner (2022), in a study conducted with professional football players, reported that athletic mental energy was positively associated particularly with tirelessness. Similarly, Shieh et al. (2023), in a study conducted with volleyball players, reported that tirelessness, calmness, confidence, and concentration were positively associated with athletic performance. The discrepancy between the findings of the present study and those reported in the literature may be attributable to dynamic factors, such as the course of the match and mood state, as well as situational factors, such as the level of competition and competitive pressure. Athletic mental energy and burnout mechanisms may vary depending on the structural demands of the sport examined, the demographic characteristics and professional level of the sample (e.g., amateur vs. professional athletes), and the environmental and psychosocial factors to which athletes are exposed. Furthermore, the differences observed in the findings of the present study may be attributable to the unique characteristics of the sample and sport-specific dynamics.

The present study demonstrated that athletic mental energy, along with its core components of concentration, confidence, and tirelessness, played a protective moderating role in the relationship between pessimism and athlete burnout. Furthermore, higher levels of athletic mental energy were found to attenuate the adverse effect of pessimism on athlete burnout. This finding suggests that athletic mental energy may function as a psychological buffer that reduces the adverse effects of pessimism on athlete burnout in the sport context. In a study conducted with rowers, Evalista and Yunus (2024) reported that optimism, a positive psychological resource similar to mental energy, had a negative moderating effect on burnout. Lu et al. (2018) found that athletic mental energy was negatively associated with burnout and positively associated with athletes’ positive mood states and that athletic mental energy moderated the relationship between stress and burnout among athletes. Arındı et al. (2025) found that, among active athletes, higher levels of mental wellbeing were associated with increased psychological performance through optimism, whereas higher levels of pessimism negatively affected psychological performance. These findings indicate that the adverse effects of psychological risk factors, such as pessimism and sport-related life stress, on athlete burnout may be weakened by positive psychological resources such as athletic mental energy. Accordingly, athletic mental energy may attenuate the adverse effect of pessimism on burnout and function as a protective psychological resource against athlete burnout.

The findings showed that concentration had a negative moderating effect on the relationship between pessimism and athlete burnout. Chiou et al. (2020) reported that concentration, one of the cognitive components of athletic mental energy, moderated the relationship between sport-related life stress, considered a negative psychological factor similar to pessimism, and athlete burnout. Furthermore, they emphasized that concentration may serve as a positive resource in protecting athletes against adverse psychological conditions such as burnout. Chang et al. (2017) reported that, unlike positive psychological resources such as mental energy, which may have beneficial effects on burnout, athletes’ negative thoughts mediated the relationship between stress and burnout and may increase athlete burnout. Abdullah et al. (2016) found that athletes who performed more successfully in critical competitions had higher levels of concentration. Sindik et al. (2015) suggested that concentration and attentional control processes enable athletes to cope more effectively with stress by filtering the pressure and negative emotional responses to which they are exposed. This finding suggests that concentration may play a role in reducing the risk of athletic burnout. Similarly, athletes with high levels of concentration may be able to maintain their cognitive focus even when experiencing pessimistic thoughts, which may help them manage the adverse effects of stress more effectively and reduce their risk of burnout. In this regard, concentration, as one of the fundamental components of athletic mental energy, may be considered an important psychological resource in athletes’ performance processes and may be a potentially protective resource against burnout.

Another notable finding was that confidence and tirelessness, two components of athletic mental energy, had negative moderating effects on the relationship between pessimism and athlete burnout. In other words, as levels of confidence and tirelessness increased, the effect of pessimism on athlete burnout decreased. Singh et al. (2024), in a study conducted with athletes competing at local, national, and international levels, found that the effect of performance on psychological wellbeing through athletic mental energy was significant and reported that athletic mental energy mediated the relationship between performance and psychological wellbeing. In the same study, both the emotional components of athletic mental energy, such as tirelessness, and the cognitive components, such as confidence, were reported to play significant roles simultaneously in this mediating relationship. Nicholls et al. (2010) found that athletes with higher levels of self-confidence had lower levels of pre-competitive anxiety and performed better. Accordingly, the moderating effect of confidence on the relationship between pessimism and athlete burnout may be related to athletes’ psychological processes. The finding that confidence and tirelessness, together with concentration, had moderating effects in this study is consistent with the literature. According to previous research, an athlete’s confidence in their own abilities, perceived competence, and the autonomy provided by their environment may be associated with lower vulnerability to chronic stress leading to emotional and physical exhaustion. When athletes feel that they have control over the process, burnout symptoms are significantly reduced (Salsabila and Jannah, 2025; Vealey and Chase, 2008). Therefore, confidence, as a positive manifestation of athletic mental energy, may help athletes cope with sport-specific life stressors. In other words, both the cognitive components of athletic mental energy (concentration and confidence) and its affective component (tirelessness) may enhance athletes’ capacity to cope with stress, thereby weakening the adverse effect of pessimism on athlete burnout. Furthermore, considering that tirelessness is one of the components of athletic mental energy (Shieh et al., 2023), it may play a critical moderating role in the relationship between pessimism and athlete burnout. Lu et al. (2018) reported that tirelessness increased in parallel with increases in energy levels and that this positively supported athletic performance. Similarly, Lane et al. (2009) concluded that increased mental energy among athletes had positive effects on performance. Lazarus and Folkman (1984), in their theory of stress and coping, proposed that individuals’ efforts to cope effectively with the challenges they encounter play an important role in restoring physical, social, and psychological balance. Within this framework, athletes with high levels of athletic mental energy, particularly high levels of tirelessness, may be able to exert greater effort to cope with sport-specific stressors and, accordingly, may experience lower levels of burnout than other athletes.

The findings indicated that the vigor, motivation, and calmness dimensions of athletic mental energy did not have significant moderating effects in the model. Both similar and contrasting findings have been reported in the literature. Schuster et al. (2016) reported that motivation is one of the most important elements in athletes’ psychological preparation and plays a decisive role in performance. Similarly, Hsieh et al. (2024) stated that athletes with strong motivation direct their energy toward their competitive goals and maintain this motivation even when faced with adversity and challenges. Isoard-Gautheur et al. (2019) also concluded that physical activity and intrinsic motivation for physical activity had no effect on burnout and cognitive fatigue scores under conditions with low levels of occupational stress. This finding suggests that the relationships between motivation and burnout and cognitive fatigue may vary under different conditions and psychological processes. The fact that cognitive fatigue exhibits a conceptually opposite structure to the mental resources contained within athletic mental energy also suggests that these relationships may differ according to different psychological conditions. The fact that motivation did not play a significant role as a moderating variable in the relationship between burnout and athletic mental energy in the present study may be related to the characteristics of the athletes participating in the study and their purposes for participating in sport. Indeed, the fact that most of the athletes participating in the study took part in university sports competitions primarily to participate and gain sporting experience rather than to pursue high-level performance goals may be one possible explanation for why motivation did not have a moderating effect on the relationship between pessimism and athlete burnout.

The findings also showed that vigor did not have a significant moderating role. However, vigor refers to an individual’s subjective mood state associated with high arousal (Lane and Terry, 2000), and high levels of vigor support athletes in maintaining their performance during demanding training sessions and competitions. Chiou et al. (2020) reported that the vigor and calmness components of athletic mental energy had significant moderating effects. Morgan (1980) reported that Olympic athletes with higher levels of vigor were more successful than those with lower levels of vigor and experienced lower levels of negative mood states such as depression, fatigue, anxiety, anger, and confusion. The absence of a moderating effect of vigor in the present study may be explained by the fact that the sample consisted predominantly of recreational or non-elite athletes and by the possibility that vigor may operate through different psychological processes associated with athlete burnout. This suggests that the role of vigor may vary across different competitive levels and psychological conditions. Therefore, further research examining the moderating role of vigor across athletes at different competitive levels and under different psychological conditions may be warranted. Similarly, calmness, as an emotional factor, is associated with high performance. Studies have shown that athletes experience calmness and emotional stability and feel physically and mentally relaxed when they achieve their best performance (Loehr, 1984). Loehr (2005) reported that athletes who experienced feelings of calmness and relaxation during an activity generally experienced a state of flow and suggested that this helped them move away from fear of failure or performance-related anxiety, fully focus on the next competition, and become completely immersed in the preparation process. Loehr (2005) reported that athletes who experienced feelings of calmness and relaxation during an activity generally experienced a state of flow and suggested that this helped them move away from fear of failure or performance-related anxiety, fully focus on the next competition, and become completely immersed in the preparation process. Anderson et al. (2014) conducted interviews with elite Australian athletes to investigate their psychological states during their best performances and found that participants reported feeling calm and relaxed during their best performances. However, the absence of a moderating role of calmness in the present study may be related to the characteristics of the sample and the participants’ purposes for engaging in sport. Indeed, the primary purpose of university sporting competitions is to promote a sports culture among university students and encourage regular participation in physical activity rather than to emphasize performance. In this context, the lack of a moderating effect of calmness may have stemmed from the fact that some participants entered the competitions with different goals rather than high performance goals. Furthermore, it should be considered that university athletes may have different levels of competition, performance expectations, and sporting participation goals than elite or professional athletes. These differences may explain the function of athletic mental energy in relation to athlete burnout. Future comparisons of university, elite, and professional athletes may be useful to assess the generalizability of the current findings to different sporting levels.

In conclusion, the present study has demonstrated that pessimism is positively associated with athlete burnout amongst university athletes, and that athletic mental energy plays a significant moderating role in this relationship. In particular, high levels of the confidence, tirelessness, and concentration components of athletic mental energy were associated with a weaker positive relationship between pessimism and athlete burnout. This finding suggests that high levels of these psychological resources may play an important role in reducing the strength of the relationship between pessimism and athlete burnout. In this regard, alongside interventions aimed at reducing risk factors such as pessimism in programs designed to prevent athlete burnout, incorporating interventions focused on developing protective resources such as athletic mental energy may help reduce athlete burnout and support athletes’ psychological wellbeing and sustainable sporting performance.

## 5 Limitations and future research

This study contributes to the sport psychology literature by demonstrating the moderating role of athletic mental energy in the relationship between pessimism and athlete burnout among university athletes. Nevertheless, the low level of additional variance explained in moderation analyses indicates that although the moderating effects are statistically significant, their contribution to the explained variance is limited. Therefore, it should be considered that the practical implications of these moderation effects may be limited. However, several methodological limitations should be considered when interpreting the findings. First, the study employed a cross-sectional research design. Therefore, although relationships among the variables can be identified, causal inferences cannot be made. In particular, future longitudinal studies are needed to examine how the moderating effect of athletic mental energy on the relationship between pessimism and athlete burnout changes over time. Furthermore, experimental studies examining the effects of psychological skills training or intervention programs designed to enhance athletic mental energy may provide stronger evidence regarding potential role of this variable as a psychological buffer.

Second, the data used in this study were collected from the same participants during a single data collection period using self-report measures. Although the measurement instruments demonstrated acceptable levels of reliability, this data collection approach may lead to measurement errors associated with common method bias and social desirability bias arising from participants’ tendency to provide socially acceptable responses.

Third, the study sample consisted exclusively of university athletes who participated in the Turkish University Athletics Championships. Indeed, most of these athletes participated in the competitions primarily for recreational and social purposes. Furthermore, according to the competition regulations, only athletes born in 1997 or later were eligible to compete, which resulted in the study sample being limited to university athletes aged 18–28 years. This may limit the generalizability of the findings to athletes from different sports disciplines, athletes with different performance levels (e.g., elite athletes), athletes from different age groups (e.g., adolescent athletes), and athlete populations from different cultural contexts. Therefore, future studies involving athletes from different sports disciplines, age groups, and performance levels, as well as from different geographical regions and countries, may strengthen the generalizability of the findings and contribute to evaluating the validity of the results across different athlete populations.

Fourth, although the moderating role of athletic mental energy was examined in this study, athlete burnout is a multidimensional phenomenon, and the current model does not encompass all variables that may influence burnout. However, the fact that individual and athletic characteristics such as age, gender, sports experience, and achievement level were not considered as control variables in the moderation model limits the interpretation of the findings by taking into account the possible effects of these characteristics. In future research, in addition to including individual and athletic characteristics such as age, gender, sports experience, national team experience, and achievement level as control variables in the model, the inclusion of individual and environmental variables such as psychological resilience, self-efficacy, coping strategies, perceived stress, psychological flexibility, motivational climate, coach-athlete relationship, social support, and training load may contribute to a more comprehensive explanation of the psychological mechanisms underlying athlete burnout. Furthermore, the use of advanced statistical approaches, such as structural equation modeling or longitudinal moderation models, may allow for a more detailed examination of the direct and indirect relationships among the variables.

In addition to future research conducted in light of these limitations, future interventions could also consider developing practices and intervention programs aimed at reducing the negative effects of pessimism among athletes, preventing athlete burnout, and enhancing athletes’ mental energy. In this context, sport psychologists, as well as coaches and performance specialists, have important responsibilities. Coaches and performance specialists may contribute to reducing pessimistic tendencies and preventing the development of athlete burnout by creating training environments that support athletes’ psychological wellbeing and strengthen positive cognitive processes. Sport psychologists may reduce athletes’ risk of burnout, support their psychological wellbeing, and help them sustain their performance by implementing psychological skills training and similar psychological intervention programs aimed at enhancing the components of athletic mental energy, including vigor, confidence, motivation, tirelessness, concentration, and calmness. Furthermore, regularly monitoring athletes’ mental energy and burnout levels throughout the season may contribute to the early identification of athletes at risk and the planning of appropriate psychological support programs. Sports federations, sports organizations, and clubs, which play an important role in determining policies related to athlete development and the enhancement of athletic performance, should develop institutional policies that support athletes’ psychological development, promote intervention programs aimed at enhancing athletic mental energy and other positive psychological characteristics, and support the adoption of holistic strategies for integrating these practices into training processes. Such practices may contribute to reducing the risk of athlete burnout and sustaining high levels of athletic performance.

## Data Availability

The original contributions presented in the study are included in the article/supplementary material, further inquiries can be directed to the corresponding author.
